# Endoscopic subserosal dissection for early gastric cancer with severe fibrosis attributable to gastric ulcer scar

**DOI:** 10.1016/j.vgie.2024.12.012

**Published:** 2025-01-07

**Authors:** Shunya Takayanagi, Ken Ohata, Darshan Parekh, Yoshiaki Kimoto, Yuki Kano, Kohei Ono, Hideyuki Chiba, Yohei Minato

**Affiliations:** 1 Department of Gastrointestinal Endoscopy, NTT Medical Center Tokyo, Tokyo, Japan; 2 Department of Medicine, Haukeland University Hospital, Bergen, Norway; 3 Bergen Research Group for Advanced Gastrointestinal Endoscopy (BRAGE), Haukeland University Hospital, Bergen, Norway; 4 Department of Endoscopy, Mumbai Institute of Gastroenterology, Mumbai, Maharashtra, India; 5 Department of Gastroenterology, Omori Red Cross Hospital, Tokyo, Japan

Endoscopic submucosal dissection (ESD) has become a standard treatment option for early gastric cancer.^1^ However, ESD is sometimes challenging when severe fibrosis is present. The en bloc resection rate is reportedly lower when severe fibrosis presents, posing a challenge and increasing procedure time.^2^^,^^3^ Especially in patients with *Helicobacter pylori* infection, significant fibrosis caused by unexpected ulcer scars requires an immediate change in strategy. Although traction devices have been reported to be helpful for dealing with fibrosis during ESD, their effectiveness in treating severe fibrosis has yet to be established.^4^^,^^5^ Severe fibrosis can make identifying the appropriate dissection plane difficult because the bluish and sparse submucosa is not visible. If the dissection plane is shallow to avoid perforation, the backside of the lesion can be injured. However, intentionally dissecting the deeper layers beneath the fibrosis can resect the entire lesion without damaging it (Fig. 1). This approach has been reported in treating early rectal cancer, allowing for accurate pathologic evaluation.^6^ This report presents a case of early gastric cancer with severe fibrosis caused by an ulcer scar, which was resected en bloc using endoscopic subserosal dissection (Video 1, available online at www.videogie.org).

**Figure 1:**
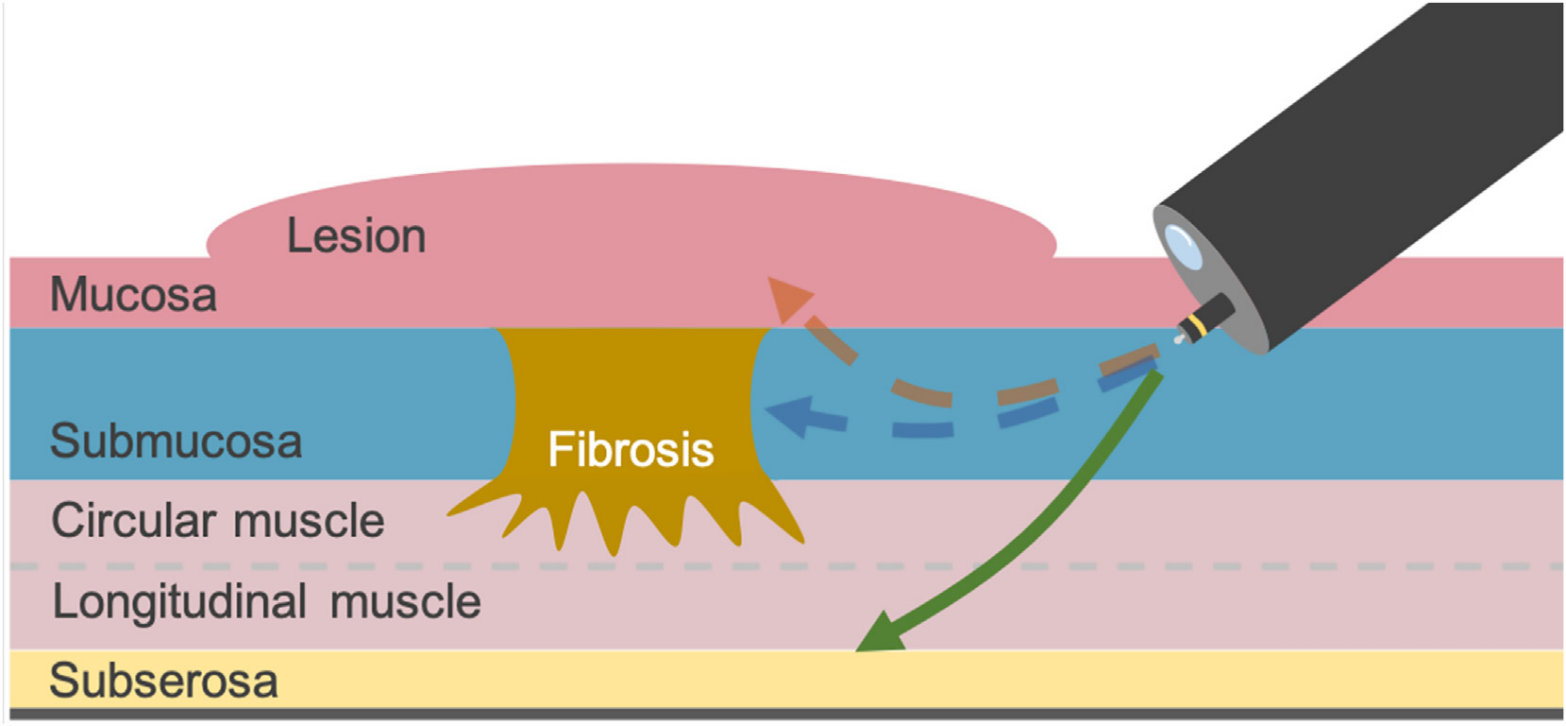
Depth of dissection for severe fibrosis in the submucosa. The fibrotic tissue should be carefully dissected (*blue arrow*), but determining the dissection plane is challenging because of the inability to create a blue submucosal cushion. Concerns about muscle injury may result in a shallow dissection plane, damaging the lesion (*red arrow*). Dissection beneath the fibrosis facilitates achieving R0 resection (*green arrow*).

## Case report

**Figure 2:**
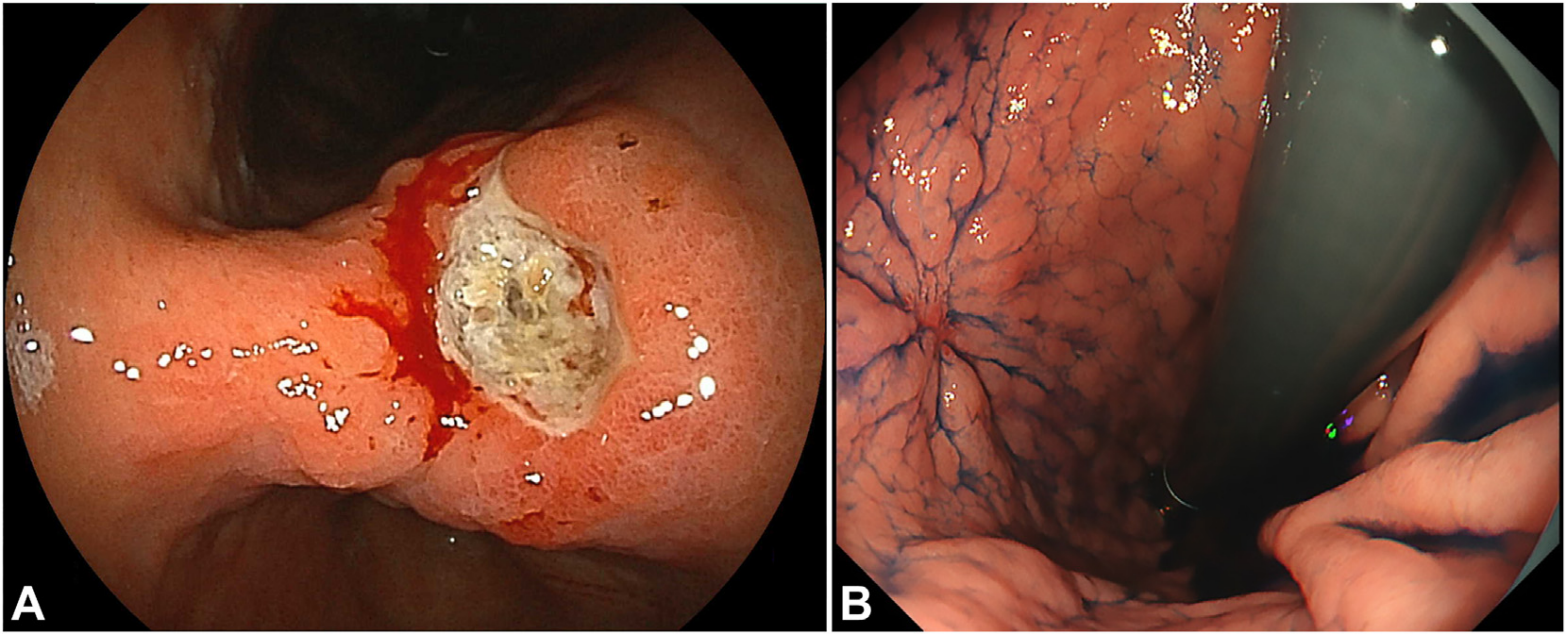
Early gastric cancer on the lesser curvature of the gastric angle. **A,** EGD reveals an early gastric cancer with ulcer on the lesser curvature of the gastric angle. **B,** Follow-up EGD confirmed that the ulcer had healed with scarring.

The patient was an 81-year-old man who underwent EGD for epigastric pain. The EGD revealed a gastric open ulcer at the lesser curvature of the gastric angle (Fig. 2A), and the biopsy showed high-grade dysplasia. After 1 month of administering oral vonoprazan, EGD confirmed that the ulcer had healed with scarring (Fig. 2B), and ESD was scheduled.

## Procedure

**Figure 3:**
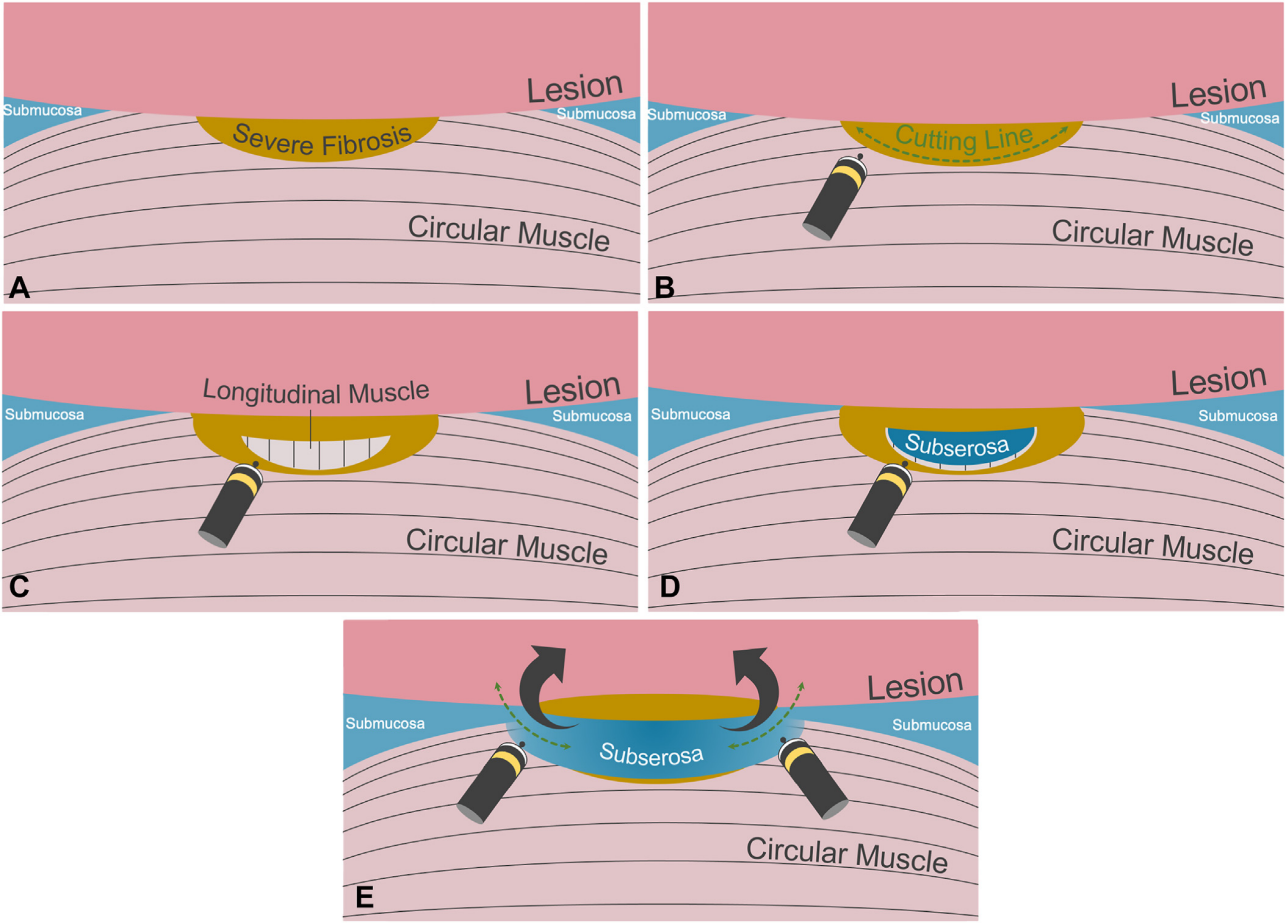
Endoscopic subserosal dissection procedure. **A,** Identify severe fibrosis in the submucosa. **B,** Dissect below the severe fibrotic tissue and the circular muscle. **C,** Expose the longitudinal muscle. **D,** Dissect the longitudinal muscle to expose the subserosa. **E,** Dissect the muscle layer to connect the submucosa with the subserosa on both sides.

Initially, the lesion was marked, and a mucosal flap was made by injecting and making a mucosal incision at the anal side of the lesion. The submucosa was highly fibrotic (Fig. 3A), and the injection with indigo carmine did not provide a blue submucosal cushion. As a result, the treatment approach was switched from ESD to endoscopic subserosal dissection. Instead of dissecting between the lesion and the muscular layer, a deliberate incision was made on the circular muscle at the edge of the severely fibrotic area (Fig. 3B). The exposed longitudinal muscle was meticulously dissected while a saline solution mixed with indigo carmine was injected from the tip of the TechKnife (Micro-Tech, Nanjing, China) (Fig. 3C). As a result, sparse blue tissue that could retain the injection solution became visible, indicating the presence of the subserosa (Fig. 3D). Both the submucosa, which lacked fibrosis, and the subserosa appeared blue. Consequently, the muscle layer between these 2 was incised, allowing access to the space beneath the lesion (Fig. 3E).

**Figure 4:**
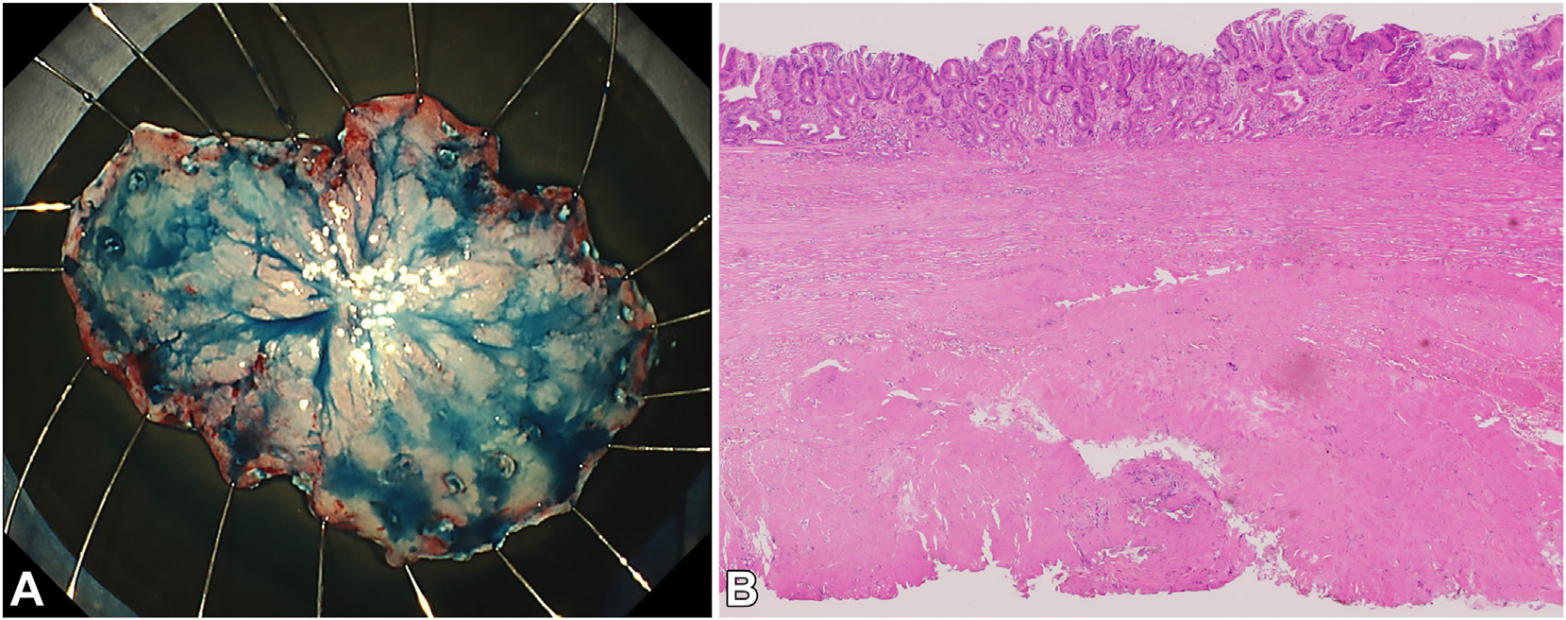
The result of the endoscopic subserosal dissection procedure. **A,** The 55-mm specimen was resected in en bloc fashion. **B,** The pathology results indicated an intramucosal well-differentiated adenocarcinoma with fibrosis (low-power view, H&E). The tumor was successfully removed along with the muscularis propria, achieving R0 resection.

Subsequently, a submucosal tunnel was created from the oral side of the lesion and passed through. Finally, the mucosa on both sides was dissected, and the whole lesion was resected en bloc (Fig. 4A).

Since the muscle defect was not very wide, using several reopenable clips (Sure Clip; Micro-Tech) was feasible. After loosening the muscle tissue through degassing, the clips were firmly inserted, ensuring a strong closure.

There were no subsequent fevers or adverse events, including perforation; therefore, no antibiotics were needed. The patient started meals on postoperative day 1 and was discharged as scheduled on postoperative day 5.

Pathology revealed an intramucosal well-differentiated adenocarcinoma with fibrosis, which was resected with the muscularis propria, achieving the R0 resection (Fig. 4B).

## Conclusions

Endoscopic subserosal dissection facilitates en bloc resection of early gastric cancer with severe fibrosis, which is challenging with ESD. Complete resection of fibrotic submucosa allows for curative and detailed pathological evaluation. Endoscopic subserosal dissection may be a viable option for the endoscopic treatment of early gastric cancer with severe fibrosis.

## Patient Consent

The patient in this article has given written informed consent to publication of the case details.

## Disclosure

All authors disclosed no financial relationships.

## Supplementary data

Video 1 Demonstration of the endoscopic subserosal dissection.
